# SNUGB: a versatile genome browser supporting comparative and functional fungal genomics

**DOI:** 10.1186/1471-2164-9-586

**Published:** 2008-12-04

**Authors:** Kyongyong Jung, Jongsun Park, Jaeyoung Choi, Bongsoo Park, Seungill Kim, Kyohun Ahn, Jaehyuk Choi, Doil Choi, Seogchan Kang, Yong-Hwan Lee

**Affiliations:** 1 Fungal Bioinformatics Laboratory, Seoul National University, Seoul 151-921, Korea; 2 Department of Agricultural Biotechnology, Seoul National University, Seoul 151-921, Korea; 3 Center for Fungal Genetic Resource, Seoul National University, Seoul 151-921, Korea; 4 Department of Plant Pathology, The Pennsylvania State University, University Park, PA 16802, USA; 5 Department of Plant Science, Seoul National University, Seoul 151-921, Korea; 6 Center for Fungal Pathogenesis, Seoul National University, Seoul 151-921, Korea

## Abstract

**Background:**

Since the full genome sequences of *Saccharomyces cerevisiae* were released in 1996, genome sequences of over 90 fungal species have become publicly available. The heterogeneous formats of genome sequences archived in different sequencing centers hampered the integration of the data for efficient and comprehensive comparative analyses. The Comparative Fungal Genomics Platform (CFGP) was developed to archive these data via a single standardized format that can support multifaceted and integrated analyses of the data. To facilitate efficient data visualization and utilization within and across species based on the architecture of CFGP and associated databases, a new genome browser was needed.

**Results:**

The Seoul National University Genome Browser (SNUGB) integrates various types of genomic information derived from 98 fungal/oomycete (137 datasets) and 34 plant and animal (38 datasets) species, graphically presents germane features and properties of each genome, and supports comparison between genomes. The SNUGB provides three different forms of the data presentation interface, including diagram, table, and text, and six different display options to support visualization and utilization of the stored information. Information for individual species can be quickly accessed via a new tool named the taxonomy browser. In addition, SNUGB offers four useful data annotation/analysis functions, including 'BLAST annotation.' The modular design of SNUGB makes its adoption to support other comparative genomic platforms easy and facilitates continuous expansion.

**Conclusion:**

The SNUGB serves as a powerful platform supporting comparative and functional genomics within the fungal kingdom and also across other kingdoms. All data and functions are available at the web site .

## Background

As the number of sequenced genomes rapidly increases, search and comparison of sequence features within and between species has become an integral part of most biological inquires. To facilitate uses of the sequenced genomes, numerous bioinformatics tools have been developed; among these, genome browser plays an essential role by providing various means for viewing genome sequences and annotated features (e.g., chromosomal position and context of individual genes, protein/nucleotide sequences, structures of exon/intron, and promoters) via graphical and text interfaces. Widely utilized genome browsers include: (i) Ensembl , which is specialized for mammalian genomics and comparative genomics [1], (ii) UCSC Genome Browser , which archives genome sequences of 30 vertebrate and 24 non-vertebrate species [2], (iii) GBrowse , a widely-used component-based genome browser [3], and (iv) Map Viewer  at the National Center for Biotechnology Information (NCBI), which covers a large number of organisms [4]. A new genome browser based on the Google map engine, called the X::Map Genome Browser [5], contains genomes of three mammalian species and is specialized for supporting microarray analyses based on the Affymetrix platform [6].

Since complete *S. cerevisiae *genome sequences were released in 1996, more than 90 fungal/oomycete species have been sequenced with many additional species being currently sequenced [7]. A few sequencing centers, such as the Broad Institute  and the JGI , have sequenced most of the fungal genomes and provide their own genome browsers to support data visualization and utilization. Although they use standardized formats, such as fasta and gff3, for data presentation and distribution, each center uses its own data formats for sequences, annotation data, and other chromosomal information. In addition, some of the sequenced fungal genomes lack certain data, such as exon positions. These problems have hampered the integration and visualization of all available genome sequences via a single genome browser. As a solution for this problem, a group at Duke University  installed an open-source browser called the GBrowse [3] after reannotating genome sequences of 42 fungal species from multiple sequencing centers through the use of their own annotation pipeline consisting of several gene prediction programs; large scale evolutionary analyses were conducted based on the archived genomes, demonstrating the usefulness of unified and standardized data formats [8].

A large number of sequenced fungal genomes have provided opportunities to compare genome sequences and features at multiple taxon levels, revealing potential mechanisms underpinning fungal evolution and biology [8-18]; however, due to the complexity and vast scale of the resulting data, presentation of these data in an easily accessible format is challenging. To overcome this limitation, both the database construction and the pipeline/tools for comparative analyses should be carefully designed. One good example is the e-Fungi project [19], which archives genome sequences of 34 fungal and 2 oomycete species and supports various queries via the web interface. Comparative fungal genomics studies have been conducted using e-Fungi [9,11]. Yeast Gene Order Browser (YGOB; ) [20] archives genome sequences of the species belonging to the subphylum Saccharomycotina and provides a graphical gene order browser, which helps the dissection of evolutionary history of genome changes during yeast speciation [21]. Although these platforms provide useful tools and data, only certain fungal genomes are covered, and the function of user-friendly access to sequence information and graphical presentation of data are limited.

The Comparative Fungal Genomics Platform (CFGP; ) was established to archive all publicly available fungal and oomycete genome sequences using a unified data format and to support multifaceted analyses of the stored data via a newly developed user interface named as Data-driven User Interface [7]. Currently, CFGP archives genome sequences of 92 fungal and 6 oomycete species (137 different datasets) and also carries genome sequences of 55 plant, animal and bacterial species (56 datasets). Taking advantage of the data warehouse and functionalities in CFGP, several databases specialized for certain gene families or functional groups have been constructed, one of which is the Fungal Transcription Factor Database (FTFD; ) [22]. This database identified and classified all fungal transcription factors and provides a phylogenomic platform supporting analyses of individual transcription factor families [23]. In addition, Fungal Cytochrome P450 Database (FCPD; ) [24], Fungal Secretome Database (FSD; ; Choi *et al*., unpublished), Fungal Expression Database (FED; ; Park *et al*., unpublished) have been constructed or are currently being constructed. The CFGP was also used to manage high-throughput experimental data and link them to corresponding genes [25,26] and to maintain the *Phytophthora *database [27].

To support comparative genomics analyses using CFGP and offer tools for versatile data visualization, we newly developed a genome browser named as the Seoul National University Genome Browser (SNUGB; ). We chose to develop a new genome browser instead of adopting one of the existing browsers in part because the adoption required conversion of the data archived in CFGP into new formats, and the existing browsers do not support the integration of additional databases, such as the InterPro and customized homologous gene databases available through SNUGB. We also wanted to have a browser based on the architecture of CFGP and associated databases so that we would be able to quickly present updated contents in these resources and seamlessly integrate new tools for data processing, visualization, and/or utilization.

The SNUGB currently covers genome sequences and associated information for 92 fungal and 6 oomycete species (137 datasets), which is the largest among the available fungal genome browser services on the web. These 92 fungal species cover four phyla and one subphylum based on a recently revised fungal taxonomy framework [28] (Table 1, 2, and 3). It also houses genome sequences of 12 plant, 18 insect, and 3 nematode species and human genome sequences (38 datasets), to support comparison of fungal genomes with those in other kingdoms (Table 4). The taxonomy browser implemented in the SNUGB provides an easy means to access genome sequences of specific species via two ways. The SNUGB provides lists of putative orthologous genes of all fungal ORFs and a tool for comparison of genomic contexts of any orthologous genes among chosen species. In addition, SNUGB displays the InterPro terms assigned to each ORF as well as the genomic regions where expressed sequence tags (ESTs) are matched. With these functionalities, SNUGB will serve as a powerful platform supporting comprehensive fungal comparative genomics.

**Table 1:** List and characteristics of the fungal genomes belonging to the subphylum Pezizomycota archived in SNUGB.

**Species**^a^	**Size (Mb)**	**# of ORFs**	**# of Exons**	**C**^b^	**I**^b^	**E**^b^	**Source**	**Refs**
**Fungi (Kingdom)**^e^								
**Ascomycota (Phylum)**								
**Pezizomycotina (Subphylum)**								
A: *Botrytis cinerea *T: *Botryotinia fuckeliana*	42.7	16,448	43,358	N	Y	N	BI	N
*Sclerotinia sclerotiorum*	38.3	14,522	40,623	N	Y	N	BI	N
*Aspergillus clavatus*	27.9	9,121	27,959	N	Y	N	BI	[17,44]
*Aspergillus flavus*	36.8	12,604	40,971	N	Y	N	BI	[16]
*Aspergillus fumigatus *AF293	29.4	9,887	28,164	8	Y	N	TIGR	[45]
*Aspergillus fumigatus *A1163	29.2	9,929	29,094	N	Y	N	TIGR	[44]
A:*Aspergillus nidulans *T:*Emericella nidulans*	30.1	10,701	35,525	8	Y	N	BI	[14]
*Aspergillus niger *ATCC1015	37.2	11,200	34,971	N	Y	N	JGI	N
*Aspergillus niger *CBS513.88	34.0	14,086	50,371	8	Y	N	NCBI	[38]
A: *Aspergillus oryzae *T: *Eurotium oryzae*	37.1	12,063	35,319	N	Y	N	DOGAN	[46]
*Aspergillus terreus*	29.3	10,406	33,116	N	Y	N	BI	[17]
A:*Aspergillus fischerianus *T: *Neosartorya fischeri*^d^	32.6	10,403	N	N	N	N	BI	[44]
*Penicillium chrysogenum*	32.2	12,791	40,441	N	N	N	NCBI	[47]
*Penicillium marneffei*	28.5	10,638	34,306	N	N	N	TIGR	N
*Coccidioides immitis *RS	28.9	10,457	36,137	N	Y	N	BI	N
*Coccidioides immitis *H538.4	27.7	10,663	34,503	N	Y	N	BI	N
*Coccidioides immitis *RMSCC 2394	28.8	10,408	34,807	N	Y	N	BI	N
*Coccidioides immitis *RMSCC 3703	27.6	10,465	33,931	N	Y	N	BI	N
*Coccidioides posadasii *Silveria	27.5	10,125	33,520	N	Y	N	BI	N
*Coccidioides posadasii *C735	26.7	N	N	N	N	N	BI	N
*Coccidioides posadasii *CPA0001	28.7	N	N	N	N	N	BI	N
*Coccidioides posadasii *CPA0020	27.3	N	N	N	N	N	BI	N
*Coccidioides posadasii *CPA0066	27.7	N	N	N	N	N	BI	N
*Coccidioides posadasii *RMSCC 1037	26.7	N	N	N	N	N	BI	N
*Coccidioides posadasii *RMSCC 1038	26.2	N	N	N	N	N	BI	N
*Coccidioides posadasii *RMSCC 1040	26.5	N	N	N	N	N	BI	N
*Coccidioides posadasii *RMSCC 2133	27.9	N	N	N	N	N	BI	N
*Coccidioides posadasii *RMSCC 3488	28.1	9,964	33,484	N	Y	N	BI	N
*Coccidioides posadasii *RMSCC 3700	25.5	N	N	N	N	N	BI	N
*Paracoccidioides brasiliensis *Pb01	33.0	9,136	37,310	N	Y	N	BI	N
*Paracoccidioides brasiliensis *Pb03	29.1	9,264	31,468	N	Y	N	BI	N
*Paracoccidioides brasiliensis *Pb18	30.0	8,741	33,239	N	Y	N	BI	N
*Blastomyces dermatitidis*	61.8	N	N	N	N	N	WGSC	N
A: *Histoplasma capsulatum *G217B T: *Ajellomyces capsulatus *G217B	41.3	8,038	26,711^f^	N	Y	N	WGSC	N
A: *Histoplasma capsulatum *G186AR T: *Ajellomyces capsulatus *G186AR	29.9	7,454	24,562^f^	N	Y	N	WGSC	N
A: *Histoplasma capsulatum *NAm1 T: *Ajellomyces capsulatus *NAm1	33.0	9,349	32,844	N	Y	N	BI	N
A: *Histoplasma capsulatum *H143 T: *Ajellomyces capsulatus *H143	39.0	7,365	25,164^f^	N	Y	N	BI	N
A: *Histoplasma capsulatum *H88 T: *Ajellomyces capsulatus *H88	37.9	7,428	25,356^f^	N	Y	N	BI	N
A: *Arthroderma gypseum *T: *Microsporum gypseum*	23.3	8,876	28,624	N	Y	N	BI	N
*Microsporum canis*	23.3	N	N	N	N	N	BI	N
*Trichophyton equinum*	24.2	N	N	N	N	N	BI	N
*Ascosphaera apis*	21.6	N	N	N	N	N	BGM	[48]
*Uncinocarpus reesii*	22.3	7,798	24,094	N	Y	N	BI	N
*Chaetomium globosum*^d^	34.9	11,124	N	N	N	N	BI	N
*Epichloe festucae*	27.0	N	N	N	N	N	OU	N
A: *Fusarium graminearum *PH-1 T: *Gibberella zeae *PH-1	36.6	13,321	37,549	N	Y	N	BI	[37]
A: *Fusarium graminearum *GZ3639 T:*Gibberella zeae *GZ3639^c^	15.1	6,694	11,692^f^	N	Y	N	BI	[37]
*Fusarium oxysporum *f. sp. lycopersici 4286	61.4	17,608	47,051	15	Y	N	BI	N
A: *Fusarium verticillioides *7600 T:*Gibberella moniliformis *7600	41.9	14,199	39,058	N	Y	N	BI	N
A: *Fusarium solani *MPVI T:*Nectria haematococca *MPVI	51.3	15,707	48,387	N	Y	N	JGI	N
A: *Pyricularia oryzae *70–15 T:*Magnaporthe oryzae *70–15	41.6	12,841	34,189	7	Y	Y	BI	[49]
A: *Pyricularia oryzae *70–15 chromosome 7 T:*Magnaporthe oryzae *70–15 chromosome 7	4.0	1,122	3,289	1	Y	N		[50]
*Cryphonectria parasitica*	43.9	11,184	33,090	N	N	N	JGI	N
*Neurospora crassa *OR74A	39.2	9,842	27,188	8	Y	N	BI	[51]
*Podospora anserina *DSM980	35.7	10,596	24,437	9	Y	N	IGM	[52]
*Trichoderma atroviride *IMI206040	36.1	11,100	32,563	N	Y	N	JGI	N
A:*Trichoderma reesei *QM6a T: *Hypocrea jecorina *QM6a	33.5	9,129	27,891	N	Y	N	JGI	[53]
A:*Trichoderma virens *Gv29-8 T:*Hypocrea virens *Gv29-8	38.8	11,643	34,673	N	Y	N	JGI	N
*Talaromyces stipitatus *ATCC 10500	35.6	N	N	N	N	N	TIGR	N
*Verticillium dahliae *VaLs. 17	33.9	10,575	29,736	N	N	N	BI	N
*Verticillium albo-atrum *VaMs. 102	32.9	10,239	28,842	N	N	N	BI	N
*Alternaria brassicicola*	32.0	N	N	N	N	N	WGSC	N
A:*Bipolaris maydis *T:*Cochliobolus heterostrophus *C5	34.9	9,633	28,007	N	N	N	JGI	N
*Pyrenophora tritici-repentis*	38.0	12,169	32,717	N	Y	N	BI	N
A: *Septoria tritici *T: *Mycosphaerella graminicola*	41.9	11,395	30,629	N	Y	N	JGI	N
A:*Paracercospora fijiensis *T:*Mycosphaerella fijiensis*	73.4	10,327	25,289	N	Y	N	JGI	N
A: *Stagonospora nodorum *T: *Phaeosphaeria nodorum*	37.2	16,597	44,017	N	Y	N	BI	[54]

**Total**	2,844.0	637,006	1,755,655	8	43	1		

^a^A indicates anamorph name and T presents teleomorph name of fungi.

^b^C means chromosomes, I indicates InterPro, and E presents EST.

^c^Incomplete coverage of genome information

^d^Insufficient exon/intron information

^e^Taxonomy based on [28]

^f^ORFs and exons were predicted by AUGUSTUS 2.0.3 with species-specific training datasets [55].

'Y' indicates the existence of information in each field, and 'N' indicates the lack of information.

**Table 2:** List and characteristics of the fungal genomes belonging to the subphyla Saccharomycotina and Taphrinomycotina archived in SNUGB.

**Species**^a^	**Size (Mb)**	**# of ORFs**	**# of Exons**	**C**^b^	**I**^b^	**E**^b^	**Source**	**Refs**
**Fungi (Kingdom)**^e^								
**Ascomycota (Phylum)**								
**Saccharomycotina (Subphylum)**								
*Candida albicans *SC5314	14.3	6,090	6,624	N	Y	N	SGTC	[56,57]
*Candida albicans *WO-1	14.4	6,160	6,395	N	Y	N	BI	N
*Candida dubliniensis*^d^	14.5	6,027	N	N	N	N	SI	N
*Candida glabrata *CBS138	12.3	5,165	5,249	N	Y	N	CBS	[58]
A: *Candida guilliermondii *T: *Pichia guilliermondii*	10.6	5,920	5,935	N	Y	N	BI	N
*Candida lusitaniae*	12.1	5,941	5,956	N	Y	N	BI	N
*Candida parapsilosis*	13.1	5,733	5,733	N	Y	N	BI	N
*Candida tropicalis*	14.7	6,258	6,292	N	Y	N	BI	N
*Candida tropicalis*^f^	2.1	N	N	N	N	N	GS	[59]
*Ashbya gossypii*	8.8	4,717	4,943	7	Y	N	NCBI	[60]
*Debaryomyces hansenii*	12.2	6,354	6,710	7	Y	N	CBS	[58]
*Debaryomyces hansenii*^f^	2.3	N	N	N	N	N	GS	[61]
A: *Candida sphaerica *T: *Kluyveromyces lactis*	10.7	5,327	5,457	N	Y	N	GS	[58]
A: *Candida sphaerica *T: *Kluyveromyces lactis*^f^	5.1	N	N	N	N	N	GS	[62]
A: *Candida kefyr *T:*Kluyveromyces marxianus*^f^	2.0	N	N	N	N	N	GS	[63]
*Kluyveromyces polysporus *DSM70294	14.7	5,367	5,524	N	Y	N	SIG	[64]
*Kluyveromyces thermotolerans*^f^	2.2	N	N	N	N	N	GS	[65]
*Kluyveromyces waltii*	10.9	4,935	5,395	N	Y	N	BI	[66]
*Lodderomyces elongisporus*	15.5	5,802	5,856	N	Y	N	BI	N
*Saccharomyces bayanus *MCYC 623	11.5	9,385	9,385	N	Y	N	BI	[13]
*Saccharomyces bayanus *623-6C YM4911	11.9	4,966	4,966	N	Y	N	WGSC	[12]
*Saccharomyces bayanus *var. uvarum^f^	4.5	N	N	N	N	N	GS	[67]
*Saccharomyces castellii*	11.4	4,677	4,677	N	Y	N	WGSC	[12]
A: *Candida robusta *S288C T: *Saccharomyces cerevisiae *S288C	12.2	6,692	7,042	16	Y	N	SGD	[68]
A: *Candida robusta *RM11-1a T: *Saccharomyces cerevisiae *RM11-1a	11.7	5,696	5,988	N	Y	N	BI	N
A: *Candida robusta *YJM789 T: *Saccharomyces cerevisiae *YJM789	12.0	5,903	6,153	N	Y	N	SI	[69]
*Saccharomyces exiguus*^f^	2.0	N	N	N	N	N	GS	[70]
*Saccharomyces kluyveri*	11.0	2,968	2,968	N	Y	N	WGSC	[12]
*Saccharomyces kluyveri*^f^	2.2	N	N	N	N	N	GS	[71]
*Saccharomyces kudriavzevii*	11.2	3,768	3,768	N	Y	N	WGSC	[12]
*Saccharomyces mikatae*	11.5	9,016	9,016	N	Y	N	BI	[13]
*Saccharomyces mikatae*	10.8	3,100	3,100	N	Y	N	WGSC	[12]
*Saccharomyces paradoxus*	11.9	8,939	8,939	N	Y	N	BI	[13]
*Saccharomyces servazzii*^f^	2.0	N	N	N	N	N	GS	[72]
*Pichia angusta*^f^	4.5	N	N	N	N	N	GS	[73]
*Pichia stipitis*	15.4	5,839	8,428	N	Y	N	JGI	[74]
*Pichia sorbitophila*^f^	3.8	N	N	N	N	N	GS	[75]
A: *Candida lipolytica *T: *Yarrowia lipolytica*	20.5	6,524	7,264	6	Y	N	CBS	[58]
A: *Candida lipolytica *T: *Yarrowia lipolytica*^f^	4.6	N	N	N	N	N	GS	[76]
*Zygosaccharomyces rouxii*^f^	4.1	N	N	N	N	N	GS	[77]
**Taphrinomycotina (Subphylum)**								
*Pneumocystis carinii*^c, d^	6.3	4,020	N	N	N	N	SI	N
*Schizosaccharomyces japonicus*	11.3	5,172	10,321	N	Y	N	BI	N
*Schizosaccharomyces pombe*	12.6	5,058	9,869	3	Y	N	GDB	[78]
*Schizosaccharomyces octosporus*	11.2	4,925	10,168	N	N	N	BI	N

**Total**	424.6	176,444	188,121	5	28	0		

^a^A indicates anamorph name and T presents teleomorph name of fungi.

^b^C means chromosomes, I indicates InterPro, and E presents EST.

^c^Incomplete coverage of genome information

^d^Insufficient exon/intron information

^e^Taxonomy based on [28]

^f^Sequences from Random Sequence Tag (RST)

'Y' indicates the existence of information in each field, and 'N' indicates the lack of information.

**Table 3:** List and characteristics of the genomes belonging to the phyla Basidiomycota, Chytridiomycota, and Microsporidia, the subphylum Mucoromycotina, and the phylum Peronosporomycota (oomycetes) archived in SNUGB.

**Species**^a^	**Size (Mb)**	**# of ORFs**	**# of Exons**	**C**^b^	**I**^b^	**E**^b^	**Source**	**Refs**
**Fungi (Kingdom)**^e^								
**Basidiomycota (Phylum)**								
**Agricomycotina (Subphylum)**								
*Postia placenta*	90.9	17,173	116,596	N	Y	N	JGI	N
T: *Phanerochaete chrysosporium *A: *Sporotrichum pruinosum*	35.1	10,048	58,746	N	Y	N	JGI	[79]
*Coprinus cinereus*	36.3	13,544	72,887	N	Y	N	BI	N
*Laccaria bicolor*	64.9	20,614	111,290	N	Y	N	JGI	[80]
A: *Cryptococcus neoformans *Serotype A T: *Filobasidiella neoformans *Serotype A	19.5	7,302	43,325	20	Y	N	BI	N
A: *Cryptococcus neoformans *Serotype B T: *Filobasidiella neoformans *Serotype B	19.0	6,870	40,589	N	Y	N	NCBI	N
A: *Cryptococcus neoformans *Serotype D B-3501A T: *Filobasidiella neoformans *Serotype D B-3501A	18.5	6,431	40,942	N	Y	N	SGTC	[41]
A: *Cryptococcus neoformans *Serotype D JEC21 T: *Filobasidiella neoformans *Serotype D JEC21	19.1	6,475	40,811	N	Y	N	SGTC	[41]
**Pucciniomycotina (Subphylum)**								
*Sporobolomyces roseus*	21.2	5,536	39,911	N	Y	N	JGI	N
*Puccinia graminis*	88.7	20,567	95,838	N	Y	N	BI	N
**Ustilaginomycotina (Subphylum)**								
*Malassezia globosa *CBS7966	9.0	4,286	4,286	N	N	N	PGC	[15]
*Malassezia restricta *CBS7877^c^	4.6	N	N	N	N	N	PGC	[15]
*Ustilago maydis *521	19.7	6,689	11,589	N	Y	N	BI	[81]
*Ustilago maydis *FB1	19.3	6,950	10,310^f^	N	Y	N	BI	[81]
**Chytridiomycota (Phylum)**								
*Batrachochytrium dendrobatidis *JEL423	23.9	8,818	38,551	N	Y	N	BI	N
*Batrachochytrium dendrobatidis *JAM81	24.3	8,732	37,423	N	Y	N	JGI	N
**Mucoromycotina (Subphylum *incertae sedis*)**								
*Rhizopus oryzae*	46.1	17,467	57,981	N	Y	N	BI	N
*Phycomyces blakesleeanus*	55.9	14,792	71,502	N	Y	N	JGI	N
**Microsporidia (Phylum)**								
*Encephalitozoon cuniculi*	2.5	1,996	2,002	N	Y	N	GS	[82]
*Antonospora locustae*^d^	6.1	2,606	N	N	N	N	JBPC	N
**Stramenopila (Kingdom)**^e^								
**Peronosporomycota (Phylum)**								
*Phytophthora capsici*	107.8	17,414	45,661	N	N	N	JGI	N
*Phytophthora infestans*^d^	228.5	22,658	N	N	N	N	BI	N
*Phytophthora ramorum*	66.7	15,743	40,639	N	Y	N	JGI	[83]
*Phytophthora sojae*	86.0	19,027	53,552	N	Y	N	JGI	[83]
*Hyaloperonospora parasitica*	83.6	14,789	24,907	N	Y	N	VBI	N
*Pythium ultimum*	44.3	N	N	N	N	N		N

**Total**	1,241.5	276,527	1,058,878	1	20	0		

^a^A indicates anamorph name and T presents teleomorph name of fungi

^b^C means chromosomes, I indicates InterPro, and E presents EST.

^c^Incomplete coverage of genome information

^d^Insufficient exon/intron information

^e^Taxonomy based on [28]

^f^ORFs and exons were predicted by AUGUSTUS 2.0.3 with species-specific training datasets [55].

'Y' indicates the existence of information in each field, and 'N' indicates the lack of information.

**Table 4:** List and characteristics of the non-fungal genomes archived in SNUGB.

**Species**^a^	**Size (Mb)**	**# of ORFs**	**# of Exons**	**C**^b^	**I**^b^	**E**^b^	**Source**	**Refs**
**Chloroplastida (Kingdom)**^e^								
**Streptophyta (Phylum)**								
*Arabidopsis thaliana*	119.2	28,581	150,369	5	Y	N	TAIR	[33]
*Carica papaya*	271.7	N	N	N	N	N	PGSC	[84]
*Glycine max*	996.9	62,199	281,102	N	N	N	JGI	N
*Lycopersicon esculentum*^c^	39.9	8,725	29,707	N	Y	N	SOL	N
*Medicago truncatula*	278.7	38,334	122,889	8	Y	N	MTGSP	[85-87]
*Oryza sativa *var. Indica^d^	426.3	49,710	N	N	N	N	BGI	[88,89]
*Oryza sativa *var. Japonica	372.1	66,710	319,140	12	Y	N	IRGSP	[89,90]
*Populus trichocarpa*	485.5	45,555	193,687	N	Y	N	JGI	[91]
*Ricinus communis*^d^	362.5	38,613	N	N	N	N	TIGR	N
*Selaginella moellendorffii*	212.8	22,285	124,645	N	Y	N	JGI	N
*Sorghum bicolor*	738.5	36,338	165,149	11	Y	N	JGI	N
*Vitis vinifera*	497.5	30,434	149,351	19	Y	N	GS	[92]
*Zea mays*^d^	2,314.7	420,732	N	N	N	N	MGSP	N
**Metazoa (Kingdom)**								
**Arthropoda (Phylum)**								
*Apis mellifera*	235.2	11,062	71,496	N	N	N	HBGP	[93]
*Acyrthosiphon pisum*	446.6	N	N	N	N	N	BCM	N
*Bombyx mori*	397.7	21,302	82,381	N	N	N	BGI	[94]
*Drosophila ananassae*	231.0	15,276	56,595	N	N	N	FB	[95]
*Drosophila erecta*	152.7	15,324	56,924	N	N	N	FB	[95]
*Drosophila grimshawi*	200.5	15,270	56,647	N	N	N	FB	[95]
*Drosophila melanogaster*	168.7	20,923	96,745	N	N	N	FB	[96]
*Drosophila mojavensis*	193.8	14,849	55,013	N	N	N	FB	[95]
*Drosophila persimilis*	188.4	17,235	59,116	N	N	N	FB	[95]
*Drosophila pseudoobscura*	152.7	16,363	57,864	N	N	N	FB	[97]
*Drosophila sechellia*	166.6	16,884	58,584	N	N	N	FB	[95]
*Drosophila simulans*	137.8	15,983	54,294	N	N	N	FB	[95]
*Drosophila virilise*	206.0	14,680	55,005	N	N	N	FB	[95]
*Drosophila willistoni*	235.5	15,816	56,641	N	N	N	FB	[95]
*Drosophila yakuba*	165.7	15,423	59,098	N	N	N	FB	[95]
*Glossina morsitans*	205.7	N	N	N	N	N	TIGR	N
*Nasonia vitripennis*	239.6	27,957	98,570^f^	N	N	N	BCM	N
*Tribolium castaneum*	152.1	14,274	58,381^f^	N	N	N	BCM	N
**Nematoda (Phylum)**								
*Caenorhabditis elegans*	100.3	26,902	175,232	7	N	N	WB	[34]
*Caenorhabditis briggsae*^d^	108.5	20,669	N	N	N	N	WB	[98]
*Caenorhabditis remanei*	145.4	N	N	N	N	N	WB	N
**Vertebrata (Phylum)**								
*Homo sapiens *Celera assembly	2,828.4	28,057	273,999	N	N	N	NCBI	[99]
*Homo sapiens *HuRef assembly	2,843.9	27,937	273,135	N	N	N	NCBI	[100]
*Homo sapiens *NCBI Reference	2,870.8	29,319	284,553	N	N	N	NCBI	[100]
*Homo sapiens*	3,665.5	43,570	452,099	29	N	N	EM	[100]

**Total**	21,241.0	1,294,281	4,142,169	7	8	0		

^a^A indicates anamorph name and T presents teleomorph name of fungi

^b^C means chromosomes, I indicates InterPro, and E presents EST.

^c^Incomplete coverage of genome information

^d^Insufficient exon/intron information

^e^Taxonomy based on [101]

^f^ORFs and exons were predicted by AUGUSTUS 2.0.3 with species-specific training datasets [55].

'Y' indicates the existence of information in each field, and 'N' indicates the lack of information.

## Construction, content, and applications

### Data processing via an automated pipeline and the function of Positional Database

Positional information of functional/structural units that are present on individual contigs/chromosomes, such as the start and stop sites of ORFs and exons/introns, was collected from the data warehouse of CFGP and stored in the Position Database of SNUGB. New types of data, such as Simple Sequence Repeats (SSRs) on the genome, can be easily added to the Positional Database for visualization via SNUGB. Along with the positional information, for each data, data type (e.g., ORFs), primary key, and any additional information were saved into the partitioned tables, which were designed for enhancing the speed of data retrieval. Through the primary key, SNUGB can display detailed information of each datum (e.g., sequences) stored at external sources. Considering the large number of available fungal genome sequences and those that are currently being sequenced, in addition to this data standardization scheme, a standardized pipeline for data extraction and management is needed to organize the data and to ensure orderly expansion of SNUGB.

The pipeline developed for SNUGB processes each genome data set via the following steps. Firstly, once whole genome sequences are deposited in the data warehouse of CFGP, the integrity of genome information, such as the position information of functional/structural units, is inspected. Several properties of the whole genome, such as the length and the GC content, are calculated. Secondly, the GC content, AT-skew, and CG-skew are calculated via 50-bp sliding windows with 20 bp steps. Thirdly, for each gene, three types of sequence information, including coding sequences (sequences from the start to stop codon without introns), gene sequences (sequences from the start to stop codon with introns), and transcript sequences (sequences from the transcription start site to end site without intron sequence), if transcript information is available, are generated based on the genome annotation information. Fourthly, all data generated in the previous steps are transferred into the Position Database to support graphical representation of these features. Fifthly, if the genome has chromosomal map information, including genetic map and optical map, this information is converted into a standardized format and stored in SNUGB for graphical representation via Chromosome Viewer. Lastly, after subjecting all ORFs in the genome through the InterPro Scan [29], the genomic position of each domain predicted by the InterPro Scan is calculated and stored into the Position Database.

### Modular design of SNUGB facilitates its application

To facilitate the efficient implementation of SNUGB in diverse genomics platforms, a modular design was used for its application programming interface (API). Through API, a diagram showing genome features in a selected region can be created using only their chromosomal positions and display options. Four recent publications illustrate the utility of this design: T-DNA Analysis Platform (TAP; ) provides the GC content and AT skew around T-DNA insertion sites on the chromosomes of *Magnaporthe oryzae *via a mini genome browser supported by SNUGB [25]. The chromosomal distribution pattern of T-DNA insertion sites in *M. oryzae * was also displayed using SNUGB [26]. Fungal Cytochrome P450 Database (FCPD; ) [24] employs SNUGB to present the chromosomal distribution pattern and contexts of cytochrome P450 genes in fungal genomes. Two databases, FED  and FSD , utilize SNUGB for presenting the genomic context of the region matched to EST and secreted proteins, respectively. Moreover, Systematical Platform for Identifying Mutated Proteins (SysPIMP; ) [30] and Insect Mitochondrial Genome Database (IMGD; ; Lee *et al*., under revision) also adopted SNUGB for data presentation. These examples illustrate the utility of SNUGB.

### Properties of the fungal/oomycete genomes archived in SNUGB

Among the 98 fungal/oomyvete species (137 genome datasets) covered by SNUGB, 77 species (111 genome datasets; 81%) belong to the phylum Ascomycota (Table 1 and 2), and 10 species (14 genome datasets; 10%) belong to the phylum Basidiomycota (Table 3). In contrast, the phyla Chytridiomycota and Micosporidia are represented only by one (2 datasets) and two species (both belong to the subphylum Mucoromycotina), respectively (Table 3). Six oomycete genomes, derived from *Phytophthora, Hyaloperonospora*, and *Pythium *species, are available for comparison with fungal genomes (Table 3). Although oomycetes belong to the kingdom Stramenophla and show closer phylogenetic relationships to algae and diatoms than fungi [31], due to their morphological similarities to fungi, they have been traditionally grouped with fungi.

**Figure 1:**
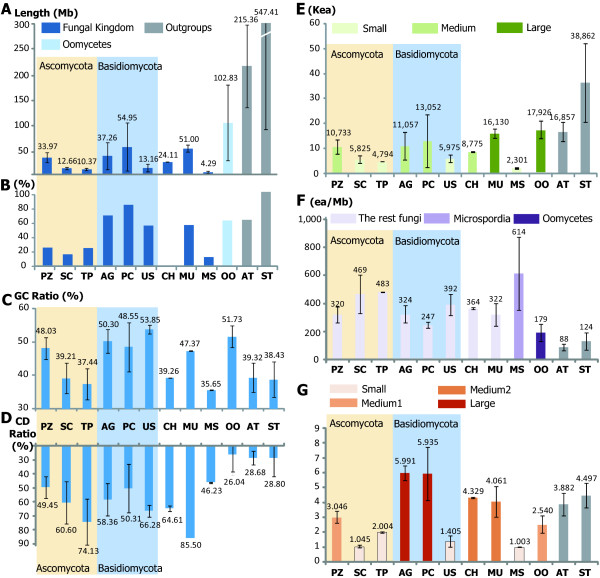
**Characteristics of the 137 fungal and oomycetes genomes archived in SNUGB**. In all graphs, the first six groups correspond to subphyla and the rests indicate phyla. Error bars indicate variation of data within each taxonomic group. The last two phyla were used as outgroup. In graphs A, E, F, and G, each color of bar indicates distinct group supported by Turkey HSD test. (A) Average genome size. (B) the ratio of variation of genome size to the average genome size. (C) Average GC ratio of each subphylum/phylum. (D) The percentage of coding regions to the genome length. (E) Average number of total ORFs. (F) The total number of ORFs per Mb (= ORF density). (G) The average exon number of each ORFs.

The datasets that cover the whole genome (121 out of the 137 datasets) were analyzed to investigate genome properties. The average size of the genomes, measured by adding lengths of all scaffolds together, is 31.42 Mb which is one-seventeenth of plant genomes (547.41 Mb in the phylum Streptophyta) and one-seventh of insect genomes (215.36 Mb in the phylum Arthropoda) (Figure 1A). The fungal/oomycete genome sizes ranged from 2.5 Mb (*Encephalitozoon cuniculi*) to 228.5 Mb (*Phytophthora infestans*); the genome of *E. cuniculi *is shorter than that of *Escherichia coli *(4.6 Mb) [32], while the genome of *P. infestans *is much larger than the genomes of *Arabidopsis thaliana *(119.2 Mb) [33] and *Caenorhabditis elegans *(100.5 Mb) [34], indicating no clear relationship between the genome size and the organismal complexity [35]. With regard to the average genome sizes in different taxon groups, the phylum Microsporidia, known as ancestral fungi, shows the smallest average size (4.28 Mb), while oomycetes show the largest at 102.83 Mb (Figure 1A). In the phylum Basidiomycota, which is large and very diverse, the degree of difference in average genome sizes within each of the represented subphyla is highest in the fungal kingdom: the ratios of standard deviation to the average length in three subphyla Agricomycotina, Pucciniomycotina, and Ustilaginomycotina are 71.95%, 86.93%, and 57.46%, respectively (Figure 1B). The subphylum Pucciniomycotina displays the largest size with large variation (Figure 1A and 1B), while two subphyla Saccharomycotina and Taphrinomycotina belonging to the phylum Ascomycota exhibit the relatively low degree of variations (Figure 1B), probably because only closely related species have been sequenced. Although the average genome sizes varied from group to group, ANOVA and TukeyHSD tests (P < 0.05) showed only the difference between fungi and oomycetes was significant (Figure 1A). The GC content of fungal genomes ranges from 32.523% (*Pneumocystis carinii *in subphylum Taphrinomycotina) to 56.968% (*Phanerochaete chrysosporium *in the subphylum Agricomycotina), while the GC content of plant and insect genomes ranges from 29.638% to 46.850% (Figure 1C). Although the coding regions exhibit higher GC contents than the rest of the genome, there is no relationship between the proportion of ORFs on the genome and the GC content of the whole genomes (linear regression; R^2 ^= 0.04; Figure 1C and 1D).

The number of total proteins encoded by each organism was once considered to reflect organism's characteristics [36]. Based on the size of total proteomes, all sequenced fungal and oomycete species were divided into three groups: The medium group contains the subphylum Pezizomycotina in Ascomycota and the subphyla Agricomycota and Puccinomycotina in Basidiomycota, the small group includes three subphyla Saccharomycotina, Taphrinomycotina, and Ustilagomycotina and the phylum Microsporidia, and the large group has the subphylum Mucoromycotina and the phylum Oomycota (ANOVA and TukeyHSD; P < 0.05; Figure 1E). This grouping shows that the number of total ORFs does not correlate with taxonomic positions at the phylum level, however, at the subphylum level, the correlation was high. For example, subphyla Saccharomycotina and Taphrinomycotina can be distinguishable from Pezizomycotina based on this character. The ORF density classified the sequenced species into three distinct groups, Oomycetes, Microsporidia and the rest, through ANOVA and TukeyHSD test (P < 0.05; Figure 1F). Taken together, these three indicators can be used to divide fungal subphyla/phyla. For example, the subphylum Pezizomycotina shows the medium-level of ORF number and ORF density, while the subphylum Saccharomycotina displays the low-level of ORF number but its ORF density is comparable to that of the subphylum Pezizomycotia. Both the number of ORFs and the ORF density are high for oomycetes, exhibiting a pattern different from fungi.

The number of exons per ORF was investigated, resulting in four groups (ANOVA and TukeyHSD test; P < 0.05; Figure 1G). With the exception of the subphylum Ustilagomycotina, the phylum Basidiomycota exhibits the highest number (~6). The subphyla Saccharomycotina and Mycoromycotina show the lowest value (nearly 1), indicating that almost all their genes do not have introns.

### Comparison of genome sequences of multiple isolates within species

For 14 fungal species, two or more strains have been sequenced (Table 5). For some species, such as *Fusarium graminearum*, additional isolate(s) were sequenced only at a low coverage (e.g., 0.4× coverage for the second strain of *F. graminearum*); however, even such low-coverage provided some insights into the evolution of pathogenicity in this important cereal pathogen [37]. Except *Aspergillus niger*, *Histoplasma capsulatum, and Paracoccidioides brasiliensis*, all strains within same species showed less than 1 Mb variation in genome sizes (Table 5). It is possible that the 3.2 Mb difference between two *A. niger *strains is in part due to different sequencing coverage: the coverage of ATCC1015 was 8.9× while CBS513.88 was 7× [38]. The differences among three *P. brasiliensis *genomes, ranging from 29.1 Mb to 33.0 Mb, may reflect their distinct phylogenetic positions [39]. The differences among five *H. capsulatum *genomes may be due to a combination of different levels of sequencing coverage  and different geological origins [40]. Three isolates of *H. capsulatum *and *P. brasiliensis *showed approximately 1% difference in the GC content, whereas the degree of GC content variation among 11 strains of *Coccidioides posadasii *was only 0.5%. Four *Cryptococcus neoformans *strains, representing three different serotypes (A, B and D), showed around 0.3% variation in the GC content, and within a serotype (two serotype D strains) the difference was only 0.043% [41]. Isolates of *Candida albicans*, *Saccharomyces bayanus*, and *Batrachochytrium dendrobatidis *showed only 0.01% variation in the GC content. These intraspecific variations of genome properties can be compared in detail via SNUGB.

**Table 5:** Basic properties of different strains of fungal genomes deposited in SNUGB.

**Species**	**# of Strains**	**Genome size (Mb)**	**GC content (%)**
**Fungi (Kingdom)**			
**Ascomycota (Phylum)**			

**Pezizomycotina (Subphylum)**			
*Aspergillus fumigatus*	2	29.3 ± 0.1	49.672 ± 0.178
*Aspergillus niger*	2	35.6 ± 2.3	50.365 ± 0.012
*Coccidioides immitis*	4	28.3 ± 0.7	46.529 ± 0.514
*Coccidioides posadasii*	11	27.2 ± 0.9	46.839 ± 0.537
*Histoplasma capsulatum*	5	36.2 ± 4.7	43.400 ± 1.859
*Paracoccidioides brasiliensis*	3	30.7 ± 2.0	43.868 ± 0.930
*Fusarium graminearum*^a^	2	36.6	48.283
**Saccharomycotina (Subphylum)**			
*Candida albicans*	2	14.4 ± 0.1	33.462 ± 0.010
*Saccharomyces cerevisiae*	3	11.9 ± 0.3	38.252 ± 0.090
*Saccharomyces bayanus*	2	11.7 ± 0.3	40.196 ± 0.011
*Saccharomyces mikatae*^b^	2	11.1 ± 0.5	37.920 ± 0.315
**Basidiomycota (Phylum)**			
**Agricomycotina (Subphylum)**			
*Cryptococcus neoformans*	4	19.2 ± 0.2	48.251 ± 0.316
**Ustilaginomycotina (Subphylum)**			
*Ustilago maydis*	2	19.7 ± 0.0	53.995 ± 0.045
**Chytridiomycota (Phylum)**			
*Batrachochytrium dendrobatidis*	2	24.1 ± 0.3	39.261 ± 0.011
**Chloroplastida (Kingdom)**			
**Charophyta (Phylum)**			
*Oryza sativa*	2	399.2 ± 38.4	43.530 ± 0.046
**Vertebrata (Phylum)**			
**Vertebrata (Phylum)**			
*Homo sapiens*	4	3,052.2 ± 409.3	40.878 ± 0.042

^a^One of strains are incomplete whole genome sequences, so that standard deviation of genome length and GC content are not calculated.

^b^Same strain but different version of assembly

### Update of SNUGB

The number of on-going fungal genome sequencing projects is approximately 40 . 37 strains of *S. cerevisiae *and 25 strains of *S. paradoxus *were already sequenced and released by the Sanger institute , indicating that more than 100 additional fungal genomes will be available soon. Next generation high throughput sequencing technologies, such as GS Flx, Solexa, and SOLiD [42,43], will further accelerate the rate of fungal genome sequencing, emphasizing the importance of frequently updating SNUGB. With the aid of the developed pipeline, SNUGB will be updated whenever new fungal genome sequences have been publicly released with annotation information. A notice for updated genomes will be posted on the SNUGB web site.

## Functions and tools

### Taxonomy browser

**Figure 2:**
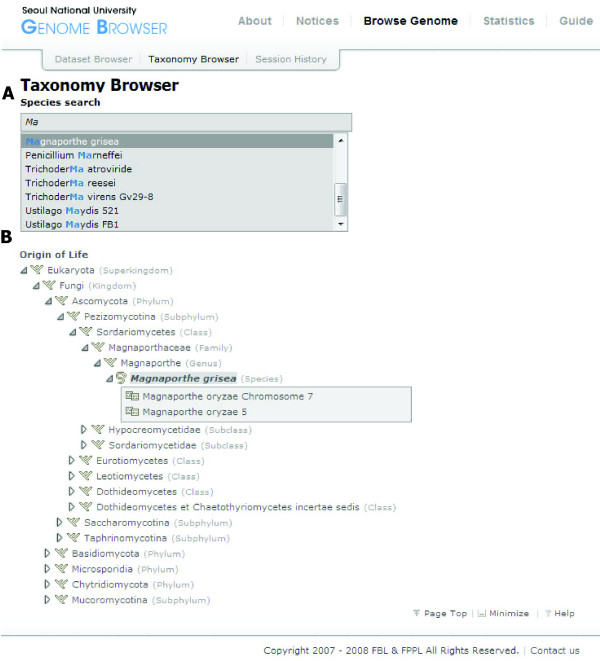
**Taxonomy browser**. A screenshot of data generated using Taxonomy browser is shown. (A) Search interface by species name shows a list of species along with inserted string. (B) Taxonomical tree shows a lineage of the chosen species and its genome datasets deposited in SNUGB.

To support selection of species of interests based on their taxonomic positions, a web-based tool, named as the taxonomy browser, was developed. Considering an anticipated increase in comparing genome sequences and features across multiple species to investigate evolutionary questions at the genome scale, such a tool is necessary to provide an overview of the taxonomic positions of the sequenced species and their evolutionary relationships with other fungi to users of SNUGB and to assist them in selecting appropriate species for comparative analyses. The taxonomy browser provides two methods for accessing the data archived in SNUGB, one of which is text-search using species name (Figure 2A). When a user begins typing a species name in the text box, the full name will be completed automatically to assist a quick search of species. The other method is using the taxonomical hierarchy (i.e., tree of life). When a user clicks a specific taxon (e.g., phylum), taxonomy browser will present all subgroups within the chosen taxon for further selection (Figure 2B).

### Chromosome viewer and Contig/ORF browser

**Figure 3:**
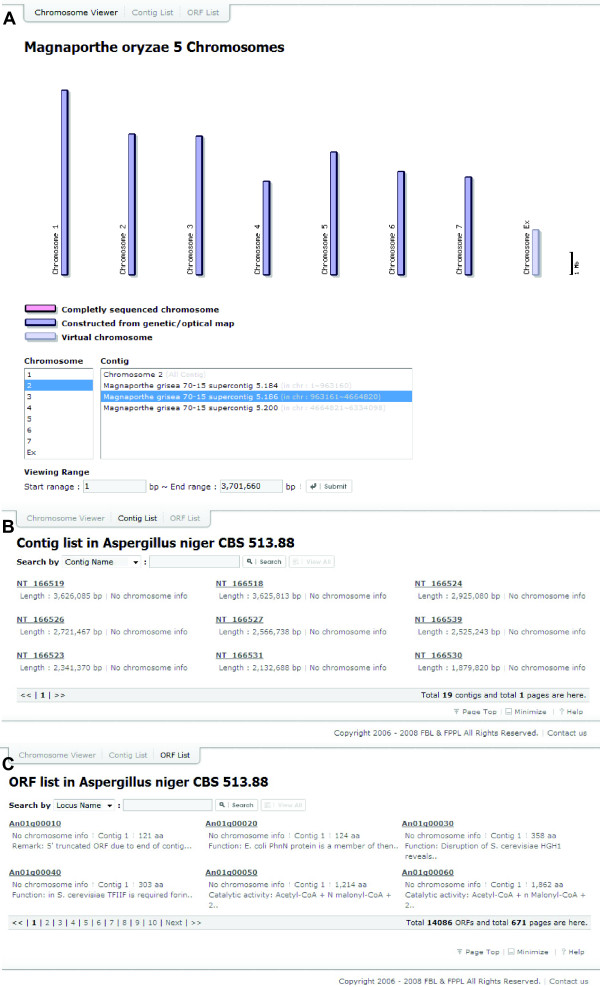
**Chromosome viewer, Contig Viewer, and ORF Viewer**. (A) The chromosome viewer displays seven chromosomes of *M. oryzae *with a size indicator at the right side. At the bottom, the interface allows for jumping directly to a specific region by selecting chromosome/contigs and its position. (B) The contig viewer provides a list of contigs with its length. Through this interface, contigs can be searched by name. (C) The ORF viewer presents the names and lengths of ORFs with search function.

Three different methods can be used to access genomic information. For those with chromosomal map data (21 species), their chromosomal maps can be displayed via Chromosome viewer (Figure 3A). The following color scheme was used to denote the level of completeness: i) chromosome constructed using genetic or optical map data (with gaps) as blue (Chromosomes 1 to 7 of *M. oryzae*; Figure 3A), ii) chromosome map based on a combination of sequences and genetic/optical map data as pink (e.g., chromosomes of *A. niger*), and iii) unassigned contigs (labeled as Chromosome Ex of *M. oryzae*; Figure 3A) as light blue. For the species without chromosomal map information, SNUGB provides the contig and ORF browsers, which display the name of contig and ORFs, respectively, and allow users to search them using their names (Figures 3B and 3C).

### Graphical Browser with six different display formats

**Figure 4:**
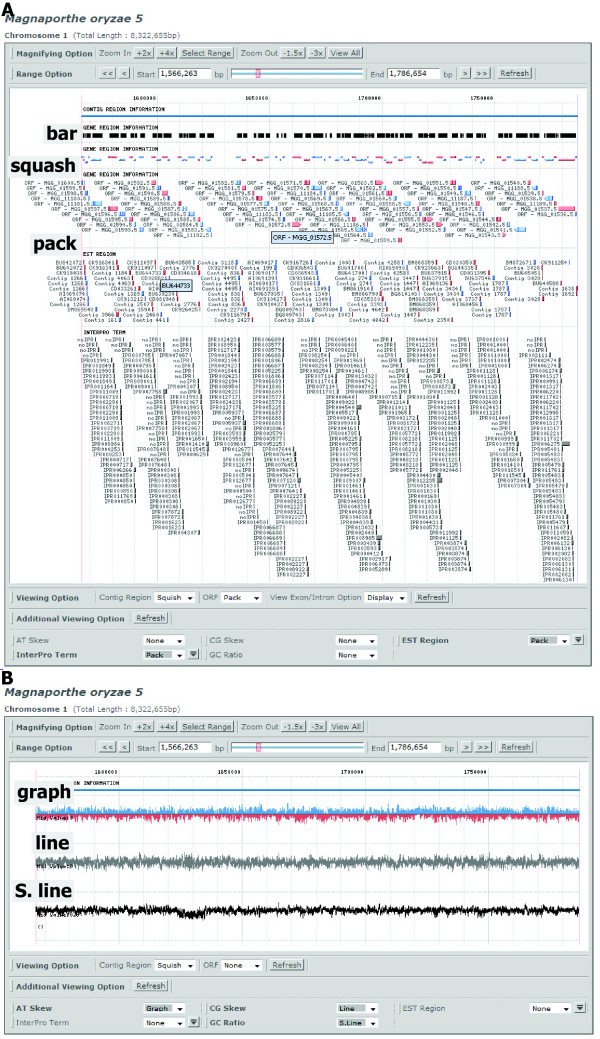
**Six different display methods of the genome content and properties via Graphical browser**. (A) The graphical browser in SNUGB shows the genome context via three different formats: bar, squash, and pack. At the bottom, ORFs, ESTs, and InterPro domains on chromosome 1 of *M. oryzae *are displayed. (B) Three graphic representations, including graph, line, and Single line (S. line), of the AT-skew, GC-skew, and GC content are shown.

Gene annotation information in a selected area of chromosome or contig, such as transcripts, ORFs, and exon/intron structure, and InterPro domains [29], can be displayed through three formats: i) the 'single' format shows these features as bars; ii) the 'squish' format displays them via color-coded diagrams without description; and iii) the 'pack' format presents them as small color-coded icons with description (Figure 4A). These graphical formats were also used by UCSC Genome Browser [2]. In addition, the GC content and AT/CG skew information for individual chromosomes can be displayed via three formats: i) color-coded bar graph, ii) line, and iii) dotted lines along with a description of data (Figure 4B). For species with EST data (Table 1), the genomic region corresponding to each EST sequence can be displayed along with ORF and InterPro domains to help users identify predicted gene structure and expressed regions (see Figure 4A). Presentation of these data is supported by Fungal Expression Database .

### Table browser and Text browser

**Figure 5:**
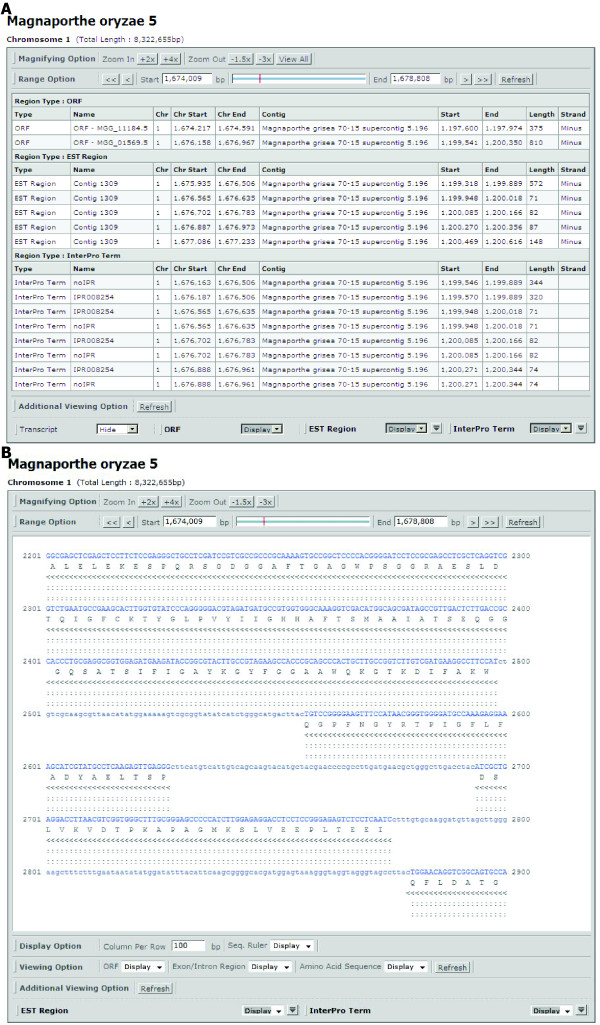
**Table and Text browsers**. (A) The table browser shows all ORFs, ESTs, and InterPro domains in a selected region as a list. (B) The text browser displays sequences showing exon/intron region as different colors and EST and InterPro domains.

Although graphical presentation of genomic features helps users view global patterns, the graphical browser does not provide sequences or a list of elements present in a chosen area. To provide such information, we developed two additional tools named as the table browser and the text browser. The table browser provides a list of the names and chromosomal/contig positions of all elements present in a selected region in the csv format, which can be opened using the Excel program (Figure 5A). The text browser provides sequences in a selected region. If ORFs exist in the region, exons and introns are presented using different colors and cases; this function is useful for designing primers and transferring selected sequences to a different data analysis environment (Figure 5B). Additionally, all InterPro domains present on each ORF are displayed as special characters under corresponding sequences so that putative functional domains can be easily recognized at the sequence level. The table and text browser can display sequences up to 50 kb.

### Kingdom-wide identification of the putative orthologues of individual fungal proteins via BLAST and comparison of the genomic contexts and properties of homologous proteins among species via the Session History function

**Figure 6:**
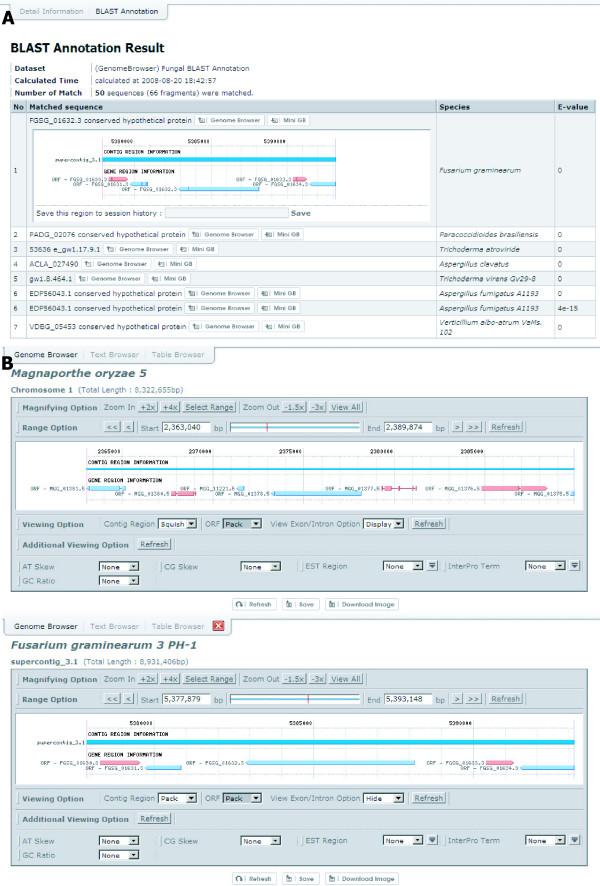
**BLAST annotation to catalog homologous proteins**. (A) A result of 'BLAST annotation' is shown with the corresponding gene names, species names, and e-values of putative homologs. 'Genome Browser' button after gene name can display the genome context of the selected gene, and 'Mini GB' button will show genome contexts of the selected gene as a smaller size to provide a quick overview, supported by MiniGB. The session can be stored by clicking the save link inside the small SNUGB image. (B) Two independent sessions showing homologs of two genes, MGG_01378.5 and FGSG_01632.3, are shown. Clicking the red button X at the bottom will hide the session.

To identify putative orthologues of individual fungal proteins, BLAST searches with each of the 924,343 fungal proteins against all proteins were performed using the e-value of 1e^-5 ^as the cut-off line. The 'BLAST annotation' tab shows a list of putative orthologues of a chosen gene product in other species with their BLAST e-values (see Figure 6A). To compare the genomic contexts around the orthologous genes between species or among multiple species, users can store the genomic contexts of the genes using the Session History function, in which the stored genomic contexts can be displayed in one screen (Figure 6B). In each session, other information, such as the GC content and InterPro terms, can also be presented to further support the comparison.

### Additional functionalities of SNUGB

The 'flexible-range-select' function allows users to select a chromosomal segment by clicking a mouse at the start site and moving it over the desired segment; the selected area will be displayed as shaded box, and the subsequent click displays an enlarged view of the selected segment (Figure 3A). Through the 'high-resolution-diagram' function, users can obtain a high-resolution image (more than 3,000 pixels in width) showing various features on a whole chromosome, such as ORFs, InterPro terms, and GC content. This image can be downloaded as image file via both the graphical genome browser and the session-storage function.

## Conclusion

The SNUGB supports efficient and versatile visualization and utilization of rapidly increasing fungal genome sequence data, as well as those from selected organisms in other kingdoms, to address various types of questions at the genome scale. Properties and features of the archived fungal genomes are available for viewing and comparison in SNUGB. The taxonomy browser helps users easily access the genomes of individual species and provides taxonomic positions of chosen species, and the chromosome map function shows the whole genome of selected species. The graphical browser, table browser, and text browser present a global view of genomic contexts in a selected chromosomal region and support analyses of sequences in the region. The 'BLAST annotation' provides lists of putatively orthologous proteins in the fungal kingdom and facilitates comparison of the genomic contexts of their genes across multiple species. The SNUGB also allows users to manage their own work histories via the SNUGB web site.

## Availability and requirements

All data and functionalities in this paper can be freely accessed through the SNUGB web site at . The source code, a set of programs, and database structure of SNUGB will be publicly released in the future after finalizing packaging of SNUGB to be opened.

## Abbreviations

PZ: the subphylum Pezizomycotina; SC: the subphylum Saccharomycotina; TP: the subphylum Taphrinophycotina; AG: the subphylum Agricomycotina; PC: the subphylum Pucciniomycotina; US: the subphylum Ustilagomycotina; CH: the phylum Chytridiomycota; MU: the subphylum Mucoromycotina; MS: the phylum Microsporidia; OO: oomycete (the phylum Peronosporomycota); AT: the phylum Arthropoda; ST: the phylum Streptophyta; BCM: Baylor College of Medicine; BGI: Beijing Genome Institute; BGM: Baylor College of Medicine; BI: Broad Institute; CBS: Center For Biological Sequences; DOGAN: Database Of the Genomes Analyzed at Nite; EM: Ensembl; FB: Flybase; GDB: GeneDB; GS: Genoscope; HBGP: Honey Bee Genome Project; IGM: Instituté de Génétique et Microbiologie; IRGSP: International Rice Genome Sequencing Project; JBPC: Josephine Bay Paul Center for Comparative Molecular Biology and Evolution; JGI: DOE Joint Genomic Institute; MGSP: Maize Genome Sequencing Project; MTGSP: Medicago Truncatula Genome Sequencing Project; OU: Oklahoma University; PGC: Procter & Gamble Co; PGSC: Papaya Genome Sequencing Consortium; SGTC: Stanford Genome Technology Center; SI: Sanger Institute; SIG: Trinity College Dublin: Smurfit Institute of Genetics; TAIR: The Arabidopsis Information Resource; VGI: Virginia Bioinformatics Institute; WB: Wormbase; WGSC: Washington University Genome Sequencing Center.

## Authors' contributions

JP and YHL planed and managed this project, KJ designed the web site, KJ, JP, BP, KA, JYC, and JHC implemented various functions to SNUGB, JP, JYC, SIK, and DC processed genome sequences, and JP, SK and YHL wrote the manuscript.
